# Preparing a financial incentive program to improve retention in HIV care and viral suppression for scale: using an implementation science framework to evaluate an mHealth system in Tanzania

**DOI:** 10.1186/s43058-021-00214-w

**Published:** 2021-09-23

**Authors:** Laura Packel, Carolyn Fahey, Atuganile Kalinjila, Agatha Mnyippembe, Prosper Njau, Sandra I. McCoy

**Affiliations:** 1 School of Public Health, University of California Berkeley, Berkeley, CA USA; 2 Health for a Prosperous Nation, Dar es Salaam, Tanzania; 3 National AIDS Control Programme, Ministry of Health, Community Development, Gender, Elderly, and Children, Dodoma, Tanzania

**Keywords:** HIV/ADS, ART, Conditional Cash Transfers, Retention in Care, Viral Suppression, Implementation Science

## Abstract

**Background:**

Viral suppression is key to ending the HIV epidemic, yet only 58% of people living with HIV (PLHIV) in sub-Saharan Africa are suppressed. Cash transfers are an effective strategy to improve retention in care, but little is known about optimization of implementation; for example, designing effective programs that integrate into existing clinic workflows. We studied implementation of an mHealth system to deliver cash transfers to support retention.

**Methods:**

We conducted a mixed-methods study assessing implementation of an mHealth cash transfer study. This was part of a larger, hybrid implementation-effectiveness randomized controlled trial evaluating cash transfers conditional on visit attendance for viral suppression among Tanzanian PLHIV initiating ART. An mHealth system using fingerprint identification and mobile payments was used to automatically disburse mobile money to eligible PLHIV. We used Proctor’s framework, assessing implementation of the mHealth system from the perspectives of PLHIV and clinicians. We analyzed mHealth system data and conducted surveys (*n* = 530) and in-depth interviews (*n* = 25) with PLHIV, clinic and pharmacy staff (*n* = 10), and structured clinic observations (*n* = 2293 visits).

**Results:**

One thousand six hundred fifty-one cash transfers were delivered to 346 PLHIV in the cash arms, 78% through mobile money. Among those in the cash arms, 81% registered their mobile money account with the mHealth system by study end, signaling high adoption. While acceptability for fingerprinting and mobile payments was high among PLHIV, interviews revealed mixed views: some had privacy concerns while others felt the system was secure and accurate, and provided some legitimacy to the clinical visits. Pharmacists praised system efficiency, but concerns about duplicative recordkeeping and added work arose. Clinic staff voiced excitement for the system’s potential to bring the cash program to all patients and simplify workflows; yet concerns about multiple systems, staffing, and intermittent connectivity tempered enthusiasm, highlighting structural issues beyond program scope. Structured observations revealed a steep learning curve; repeat fingerprint scans and manual entry declined as the system improved.

**Conclusions:**

Biometric identification and mobile payments were acceptable to most patients and staff. Fingerprinting encountered some feasibility limitations in the first months of testing; however, mobile payments were highly successful. Biometric identification and mobile payments may provide a scalable mechanism to improve patient tracking and efficiently implement financial incentives in low-resource settings.

**Trial registration:**

Name of the registry: clinicaltrials.gov

Trial registration number: NCT03351556

Date of registration: 11/24/2017

Checklists: StaRI (included with submission). Note CONSORT for cluster-randomized trials was used for the main trial but is not directly applicable to this manuscript.

**Supplementary Information:**

The online version contains supplementary material available at 10.1186/s43058-021-00214-w.

Contributions to the literature

- This study uses a mixed-methods approach to study implementation of an mHealth cash transfer intervention as part of a larger implementation-effectiveness trial designed to optimize the implementation strategy for improving retention in care and viral suppression among ART initiates
- Our mixed-methods approach uncovered unanticipated benefits and challenges to implementing cash transfers in a clinical setting — important insights for program directors and policy makers in low-resource settings considering cash transfer programs
- Our results illustrate how an mHealth system can streamline implementation of a cash transfer program, increasing the potential for sustainability and scale.

## Background

For people living with HIV (PLHIV), antiretroviral therapy (ART) is an effective strategy to clinically suppress the virus, providing the dual benefit of improved health and vastly decreased risk of transmission. Despite the robust evidence, this highly effective intervention has not yet reached all that could benefit. For example, in Tanzania, of the 1.6 million people living with HIV, only 62% are virally suppressed, and of those who are currently on HIV treatment, 87% are virally suppressed [1, 2] — falling short of the 95-95-95 goals that UNAIDS has set for 2030 for which 95% of those on HIV treatment should be virally suppressed [2]. To reach these goals, new and effective implementation strategies that can put evidence into widespread practice and bring sustained HIV treatment for viral suppression to all PLHIV are needed. mHealth systems, in combination with other evidence-based implementation strategies, have the potential to facilitate reaching the 95-95-95 goals by streamlining clinic-based recordkeeping, improving care coordination, and simplifying intervention implementation.

Cash incentives are increasingly recognized as one such evidence-based implementation strategy: these programs typically provide cash (or other incentives) to participants who meet a particular condition, for example testing for HIV, thus motivating certain behaviors that result in improved health. There is a substantial evidence base demonstrating the effectiveness of cash transfer programs in improving outcomes along the HIV care continuum in low-resource settings in a research context [2–19], with few negative impacts of transfers reported [20, 21]. Despite strong evidence and the increasing prevalence of large-scale government-run cash transfer programs for social protection, few cash transfer programs to improve HIV outcomes have been scaled. One possible reason for this gap is that simplified, automated models for implementing these clinic-based programs at scale have yet to be developed and evaluated.

For a clinic-based cash transfer program contingent on visit attendance to be scalable and sustainable in resource constrained settings the delivery model must be simple. We developed an mHealth system designed to automate and simplify cash distribution through integration with mobile money providers. The mHealth system had the dual purpose of monitoring compliance (visit attendance) through biometric identification and automatically delivering cash transfers to those who were eligible, obviating the need for manual monitoring of compliance with clinic visits and manual delivery of cash [22]. While it was not part of the mHealth intervention to replace existing tracking systems in clinics, we sought to understand the feasibility of replacing and/or supplementing systems.

We conducted a mixed-methods study assessing implementation of an mHealth cash transfer intervention as part of an implementation-effectiveness randomized controlled trial. The trial evaluated two cash transfer sizes (~ $5 and ~ $10) conditional on visit attendance, with the outcome of viral suppression at 6 months among PLHIV recently initiating ART in Shinyanga, Tanzania. Results of the trial indicated that cash was effective in improving viral suppression at 6 months, especially with the larger cash amount [23]. Additionally, the intervention improved retention in care. This showed that incentives for clinic attendance do result in greater adherence to ART (i.e., patients with pills on hand are more likely to swallow them) [23, 24].

In this trial, the cash transfer *distributed through the mHealth system* is the implementation strategy that aims to improve retention on ART (evidence-based intervention) and ultimately viral suppression among new ART initiates. The actors were the clinical staff with support from the research staff in using the mHealth system. The components of the implementation strategy were registration in the mHealth system, fingerprint identification at each visit, and automated cash disbursement via mobile money.

In this manuscript, we present the results of the mixed-methods study assessing the implementation of the mHealth cash transfer. The overarching goal of the evaluation was to understand from both clinician and patient perspectives the level of fit of the mHealth system with current health systems in Tanzania, and thus to provide recommendations for bringing the system to scale, consistent with Tanzania’s country-level strategic goals relating to mHealth [25].

## Methods

### Setting

This study took place within four HIV care and treatment clinics located in the Shinyanga region of Tanzania. Shinyanga is located in the Lake Zone region of Tanzania, a rural part of the country where over 4.5 million people live in poverty (32–35% of the population) [26]. Adult HIV prevalence in Shinyanga is 5.9% (4.7% nationally); among those living with HIV in Shinyanga, approximately 40% are virally suppressed (62% nationally) [27].

### Intervention

This paper reports on a mixed-methods, implementation science study nested within a randomized controlled trial, which has been described in detail elsewhere [23]. Briefly, from April to December 2018, 530 adult PLHIV who were initiating ART were enrolled and randomized to three arms: standard of care, smaller cash transfer intervention (~ $4.50 US) and larger cash transfer intervention (~ $10 US). Participants in the intervention groups were provided with the cash transfer contingent on monthly appointment attendance during the first 6 months of HIV treatment; the primary outcome was viral suppression as measured at the 6-month appointment. Attendance monitoring and cash transfers were implemented using a tablet-based mHealth system.

### Description of the mHealth system

In collaboration with a Tanzania-based technology firm, we created an mHealth system with the following key components: (1) pharmacy-based monitoring of patient visit attendance, (2) biometric identification using fingerprinting, and (3) cash disbursement for those in the intervention group integrated with all mobile money providers in Tanzania. Individual mobile money account details were registered in the mHealth system during enrollment for participants who had access and consented to mobile payments. The mHealth system was designed to be implemented in the pharmacies co-located within HIV primary care clinics; upon picking up medication, all participants were to use the fingerprint scanner to register in the system. The system then displayed a form in which the pharmacist or research assistant entered specifics about the medication dispensed (type of ART and number of pills) as well as the next appointment date. For those in the intervention groups, upon completion of the pharmacy visit form, the cash transfer would then be automatically sent to the registered mobile phone number via mobile money (cash was available to those who did not have access to mobile money). Once received (typically within minutes), the participant receives an SMS confirming receipt of the transfer. The key design components of the system, intended functionality, intended benefits, and learnings are detailed in Table 1**.**

**Table 1:** Afya mHealth components and lessons learned

Component	Functionality	Intention	Lessons learned
Pharmacy-based monitoring patient visit attendance	Automatically logs patient attendance and allows entry of next visit date Pharmacists registers patient with biometric ID and inputs medication pickup, next appointment into the system Robust measure of retention — last stop of clinic visit, made sense for cash to be disbursed here	Track patient medication pickup in nearly real-time (robust measure of retention) Pharmacists entering data into the mHealth system to document receipt and type of medication given Overcomes key information gaps that could benefit care Obtain next visit date for accurate measurement of retention in care	**The program needs to have readily available benefits for users that are clear to clinic staff.** **Training and engagement with clinic staff as partners will be essential as their buy-in is key to successful implementation.**
Biometric ID	Identifies patient using fingerprint, logs appointment attendance for cash eligibility, provides near real-time information about visit attendance	Saves clinic time by efficiently and rigorously monitoring patient visit attendance Avoids participant misclassification that can arise from lengthy clinic IDs that must be transcribed from paper records to the database	Overall, fingerprint identification was highly acceptable to patients and clinicians and may increase the system’s perceived legitimacy. iterative improvements were required to overcome fingerprint recognition challenges. **It took a while to get this right.**
Financial incentive disbursement integrated with mobile money	Automatically sends cash for visit attendance via the mobile money provider used by the client, seamlessly incorporates any program “rules” without human error	Simplifies the logistics of disbursing cash payments Increases privacy compared to cash Auto transfer connected to fingerprint scan bolsters connection between visit attendance and cash	Overall, the mobile money disbursement was highly acceptable Some concerns about privacy and autonomy in spending. **At scale, automated cash distribution will be essential for feasible implementation of financial incentives in HIV clinics.**

Following the launch of the mHealth system in the study clinics, we made iterative improvements to overcome fingerprint recognition challenges. It should be noted that use of the mHealth system in general did require significant support from our research team, albeit more in some clinics than in others. This was by design for this phase of the trial. We are currently enrolling participants for the next phase of this work - a cluster-randomized control trial with 32 facilities as clusters across 4 regions in Tanzania [28], where we are applying a more hands-off approach where after supportive supervision, clinical staff are using the mHealth system independently.

### Participants

The implementation science study participants included a subset of PLHIV enrolled in the RCT, as well as clinical staff from the four study clinics. Details on the inclusion/exclusion criteria and recruitment process are provided elsewhere [23]. Participants in the cash groups were asked to share preferences on mobile money and a subset of participants was asked for more detailed feedback about the entire mHealth system (described below). In-depth interviews were conducted with 25 PLHIV who were selected using purposeful sampling. Participants were purposively selected such that we had equal numbers of males and females, and equal representation of both levels of cash groups. We recruited 10 clinic and pharmacy staff at the four study clinics. For clinical staff, the purpose was to have representation of all types of clinical staff involved in the study. We randomly selected participants to reach these equal distributions. Inclusion criteria for participation were working as clinical staff at one of the study clinics during study implementation, interacted with the mHealth system in the clinic, and age 18 or older. The number of participants was determined with the assumption that we would reach saturation of themes. All participants provided written informed consent prior to participation.

### Data collection — surveys

At the 6-month follow-up visit, a subset of participants were asked about two aspects of the mHealth system using an adapted version of the Health Information Technology Usability Evaluation Scale (HITUES) [29]: the biometric identification feature (*n* = 104 participants from all study groups) and the automatic mobile money disbursement (*n* = 53 participants from the intervention groups). The HITUES is divided into four domains to assess the impact, usefulness, ease of use, and user control of the system from the perspective of the patient (see supplemental Table S1 for all questions, by domain). Possible responses were on a 5-point scale of agreement, with higher scores relating to strong agreement with the presented statement. In addition, all participants in the cash groups (*n* = 346) were asked about their preferences for mobile money versus cash in hand. All surveys were conducted using Qualtrics offline surveys.

### Data collection — in-depth interviews

We conducted semi-structured in-depth interviews (IDIs) with PLHIV (*n* = 25) and clinic and clinical staff (*n* = 10). PLHIV were asked about their experience with the biometric fingerprinting system of the mHealth system and about their experience with the cash transfer (via mobile money or cash). These interviews were conducted at the 6-month follow-up visit. Clinical staff were asked about the mHealth system as a whole and their experience using it as part of clinic operations in the context of the study. Interviews with clinical staff were conducted at the end of the study. All qualitative interviews were conducted in Kiswahili and recorded, transcribed and translated into English.

### Data collection — structured observations

Four months into the study, we initiated structured observations in all of the study clinics (*n* = 2293 visits) to document functionality of the biometric identification system and the mHealth system. Data were collected on a per visit basis (not containing identifiable information), and documented using Google Forms. Data were then imported into STATA for analysis.

### Implementation outcomes

We selected outcomes guided by Proctor’s implementation science framework [30]. We explored these outcomes from both the PLHIV and clinic staff perspectives. Specifically, we triangulated data from multiple sources to evaluate the following outcomes: appropriateness, acceptability, fidelity, adoption, coverage, and sustainability. Table 2 lists each outcome, adapted definition of that measure, indicator used to assess the outcome, data source used, and relevant population, e.g., PLHIV or clinical staff. (The subsequent results section is organized by implementation science outcome and participant perspective.)

**Table 2:** Implementation science outcomes table, with population of interest indicated in square brackets

Proctor outcome	Adapted definition	Measure/indicator [population perspective]	Data source
Acceptability	The degree to which the mHealth components are considered reasonable and satisfactory given current context	% Consented to mobile money [PLHIV] % Consented to biometric ID [PLHIV] Preference for mobile money vs. cash [PLHIV] Preference for traditional paper-based system vs. biometric ID [PLHIV and Clinical Staff] Clinical staff perspectives on the mHealth system [Clinical Staff]	Baseline/endline survey with PLHIV In-depth interviews
Appropriateness	The perceived fit of the mHealth system components within the existing context	HITUES [PLHIV] Clinical staff perspectives on mHealth system [Clinical Staff]	HITUES In-depth interviews with Clinical Staff
Adoption	The level of uptake of the mHealth system components	% With mobile money linked to mHealth at endline [PLHIV] % Visits where the pharmacist was operating the mHealth system [Clinical Staff]	mHealth system data Structured observations
Fidelity	The degree to which the components of the mHealth system were implemented as intended	# Visits mHealth system not used [Clinical Staff] % Eligible receiving cash transfers [PLHIV] % Cash transfers sent through mobile money [PLHIV] % Mobile transfers requiring manual re-sending [PLHIV]	Structured observations mHealth system data
Feasibility	The degree to which the technical aspects of the mHealth system functioned as intended (technology gaps or glitches)	% With access to mobile money [PLHIV] % With mobile phones [PLHIV] % Of scans that were successful [Clinical Staff] Average number of scans required [Clinical Staff] Number/proportion of errors in mobile transfers [Clinical Staff]	Baseline/endline survey In-depth interviews mHealth data system
Sustainability	The degree to which the mHealth system might be scalable and sustainable in HIV clinics	Barriers to scale-up [Clinical Staff]	In-depth interviews

This study was approved by the University of California, Berkeley Office for Protection of Research Subjects and by the National Medical Research Institute of Tanzania. All study participants were required to provide written informed consent prior to study participation.

### Analysis

Descriptive analyses of quantitative data collected through surveys and structured observations were analyzed using STATA statistical software [31]. Qualitative data were analyzed using an inductive data analysis approach [32]; themes were documented by two coders as they emerged. The coding framework was developed iteratively. First, high-level codes corresponding to the category of questions were developed. Sub-codes were then developed based on themes that arose from the transcripts. Coding was conducted by the Tanzanian and US teams independently; coded transcripts were then compared, and discrepancies were resolved through discussion. All coding was conducted using Dedoose [33].

## Results

Table 3 shows the demographic information for all 530 PLHIV enrolled in the full trial, the subset of the 104 PLHIV (19.6%) who responded to the HITUES survey, and the subset of 25 PLHIV (4.7%) who participated in the in-depth interviews. The mean age ranged between 34 and 36 years, and across the three groups the majority of participants (330, ~ 60%) were women. Most were in a monogamous marriage, and initiated ART at WHO clinical HIV stage 1. The majority reported having access to a mobile phone at baseline (466, 88%), and slightly fewer reported having access to mobile money at baseline. By study end, 429 (81%) of participants had registered their mobile money account with the mHealth system.

**Table 3:** PLHIV participant demographics at study enrollment for the full study, those included in the IDIs, and those who were surveyed with HITUES

	Total	Included in IDIs	Included in HITUES
	***N*** = 530 (***n***, %)	***N*** = 25 (***n***, %)	***N*** = 104 (n, %)
**Mean age (years) at baseline**	36.1 (10.2)	33.8 (10.3)	35.9 (10.7)
**Sex**			
Male	200 (37.7)	10 (40.0)	39 (37.5)
Female	330 (62.3)	15 (60.0)	65 (62.5)
**Ever attended school**			
No	113 (21.3)	3 (12.0)	20 (19.2)
Yes	417 (78.7)	22 (88.0)	84 (80.8)
**Current marital status (baseline)**			
Single/never married/no partner	39 (7.4)	0 (0.0)	5 (4.8)
Unmarried, with partner	57 (10.8)	3 (12.0)	16 (15.4)
Married (monogamous)	200 (37.7)	10 (40.0)	34 (32.7)
Married (polygamous)	31 (5.8)	0 (0.0)	7 (6.7)
Widowed	45 (8.5)	1 (4.0)	9 (8.7)
Divorced	44 (8.3)	2 (8.0)	7 (6.7)
Separated	114 (21.5)	9 (36.0)	26 (25.0)
**WHO clinical stage (baseline)**			
Stage 1	281 (53.0)	19 (76.0)	58 (55.8)
Stage 2	187 (35.3)	6 (24.0)	31 (29.8)
Stage 3	59 (11.1)	0 (0.0)	13 (12.5)
Stage 4	3 (0.6)	0 (0.0)	2 (1.9)

### Acceptability

#### Fingerprint scanning: patient perspective

We found that most PHIV were comfortable with the fingerprint scanning and the mHealth system overall, especially given the growing ubiquity of biometric identification in Tanzania. Several mentioned that biometrics are the way things are moving in the country and that many businesses are already using it.

The good thing [about the fingerprinting] is about confirmation, because if the fingerprint is not yours then the system won’t confirm…I knew it was something useful in most sectors that’s why I didn’t see any problem... (Female, age 43, clinic A)

Some PLHIV felt that fingerprint scanning added not only accuracy in confirming their identity, but also legitimacy, security, and enhanced reliability in tracking visits and medication pick up.

The current system which we used to scan should continue…this system is assured…the data remain safe and in a good system. By the previous system, you can forget, you can also lose the papers… (Female, age 52, clinic B)

Participants noted that the fingerprint system simplified the appointment process and remarked that they spent less time at the clinic once they started using the fingerprinting and mHealth system. While the mHealth system did not completely obviate the need for paper files, the perception among some was that the system did streamline the clinic process and procedures. However, in contrast to those who talked about the efficiency or simplicity of the system, some participants brought up that they felt the system caused delays at the clinic because of its dependency on a reliable network connection, and delays related to staff who were not proficient with the system.

…some pharmacists were good and some I can say didn’t know how to use it [mHealth system]… if you meet with the one who is well experienced you don’t spend a lot of time but another might tell you the system is not working because they don’t know how to use it. (Male, age 27, clinic A)

#### Mobile money: patient perspective

Using the survey and enrollment data, we found high levels of acceptability of the mobile money system and the fingerprint registration process among PLHIV. Nearly all (99%) of those who had access to a mobile bank account consented to automatic cash disbursement through mobile money. Further, we found that 98% of eligible participants consented for the study (consenting included fingerprint scanning).

In both IDIs and the structured survey, we asked participants (in the cash groups) about their experiences receiving the cash and, regardless of how they received the cash during the study, whether they would in general prefer to receive the transfers in cash or delivered through mobile money. The interviews point to considerable variation of preferences for delivery in cash as compared to delivery as mobile money, and in the rationale cited for these preferences. Specifically, one theme that emerged related to the acceptability of mobile money included accuracy, safety, and reliability:

I can say there is accuracy because when money is sent from the machine to my phone it means there is a report that will be sent…there is no security in giving someone cash in hand because the sender may not get the money to the intended person… (Female, age 30, clinic A)

A second theme that was discussed related to control over spending and ability to save money*.* Interestingly, several PLHIV talked about how they used delivery via mobile money as a way to save or control spending; they were less likely to simply spend the cash on the way home if the money was in their phone versus in their pocket, for example.

…another thing is when you have the money in hand you may end up spending all of it in things that are of no importance., But when it’s in the phone you can leave the clinic …and the money remains in your phone…So keeping it there helps, it’s like a small bank, your personal bank. (Female, age 23, clinic A)

A third theme that was brought up by PLHIV related to privacy concerns. Some noted that mobile money offered increased privacy compared with receiving cash; in particular, mobile money was discreet enough that their participation in the study could remain private and was not revealed to others attending the clinic, for example. However, others noted that they felt that the cash was more private that the mobile money option — some expressed concern that someone would see the message on the phone that appears when the money has been delivered and know they received money. Related to this, some women noted that cash allowed for more decision-making power — for example, they may share a phone with their partner, and if the money is delivered through the phone, their partner will see the SMS message, and they will no longer be able to decide how to spend the money on their own. In addition, others noted that they were concerned that the SMS message alerting them that the mobile money had been delivered might reveal something about their HIV status or that they were participating in a study. One female participant explains why she prefers to receive cash:

…I live with my husband there at home and he is not supportive to children, sometime my children may need a small amount of money to use at school but he will not help…so [the cash] helps me, but if sent through the phone, he must know it when he read the messages in my phone and he will start to question and this will be a problem. (Female, age 44, clinic C)

Other themes that emerged related to preference for cash over mobile money included concerns about the technology infrastructure (e.g., the unreliable nature of the network) and fees related to the use of mobile money services.

[With cash] You don’t get a disturbance of going to the M-Pesa where there will be some deductions, when given at hand; you just put it in your pocket and leave. (Female, age 33, clinic A)

#### Clinical staff perspective

To understand the level of acceptability of the mHealth system among clinical staff, we evaluated themes emerging from the in-depth interviews with clinicians, pharmacists, and pharmacy staff. We defined acceptability from the clinical perspective as the degree to which the mHealth components are considered reasonable and satisfactory given current clinical working environment. One of the themes that emerged in discussing the mHealth system with the clinical staff related to the perception that use of the system would result in additional work for the staff.

…on the side of staff, it [mHealth system] will be something new…it will be a new task which used not to be there…it can bring like a sort of resistance to change but with time they will cope. (CTC In Charge, clinic B)

Others, however, noted that the system was easy to use once they had adequate training, that it simplified their work and helped with managing patient flow within the clinic.

At first, I was afraid to use this system and I told them that I can’t work with it and they told me that you will know it, so they directed me how to use it and I can now use it. (Clinic Staff, clinic D)

Finally, some clinicians noted that many systems come and go as part of research or government programs, and the clinics often do not see any lasting benefit.

### Appropriateness

#### Patient perspective

We used the HITUES to assess the appropriateness of the automated mobile money system and the fingerprint components of the mHealth system among PLHIV (Table 4). We adapted the HITUES to explore domains of appropriateness, defined here as the perceived fit of the mHealth components (mobile money, fingerprinting) within the existing clinical care context for PLHIV. Those four domains included impact, usefulness, ease of use, and user control (see supplementary materials for full question text). The overall average score for the scale as it related to using fingerprinting for biometric identification was 4.1 (out of a total possible 5.0). Scores for the fingerprinting showed even less variation than those for the automatic mobile money disbursement, ranging from 4.0 (usefulness domain) to 4.2 (user control domain).

**Table 4:** Mean scores of PLHIV on the Health Information Technology User Evaluation Scale applied to fingerprint identification and mobile money

HITUES domain	Mean	Std error	Lower 95	Upper 95
**Fingerprint ID (*n*= 104)**				
Impact	4.10	0.07	3.96	4.24
Usefulness	4.01	0.07	3.87	4.15
Ease of use	4.16	0.07	4.03	4.30
User control	4.21	0.07	4.07	4.34
Fingerprint overall average	**4.10**	**0.06**	**3.98**	**4.21**
**Mobile money (*n*= 53)**				
Impact	4.32	0.11	4.10	4.54
Usefulness	4.18	0.08	4.02	4.34
Ease of use	4.32	0.09	4.15	4.49
User control	3.80	0.06	3.67	3.93
Mobile money overall average	**4.20**	**0.08**	**4.04**	**4.36**

The overall average score for the scale as it related to the automatic mobile money disbursement (compared to receiving cash in hand) was 4.2 out of a possible 5, indicating that participants found the system both acceptable and useful, with minimal variation by domain. The impact domain had the highest average score (4.3 out of a possible 5 points).

#### Clinical staff perspective

We explored appropriateness of the mHealth system with clinical staff through the in-depth interviews. We defined appropriateness as the perceived fit of the mHealth system components within the existing clinic context. Discussions converged around the following themes: technical difficulties, staff shortages and staff turnover, facilitation of patient follow-up, benefits for the patients, and spillover effects for patients not enrolled in the study.

In terms of technical difficulties, some staff expressed frustration, as the system did not always work, and was dependent on having a reliable network connection.

…if the customer put his/her finger prints, the system fails to show recognition, you may try the left hand but it does not respond, the same with the right hand also, so you have to use the ID number, so this is what challenges me. (Clinic Staff, clinic D)

Other clinicians discussed the difficulties of implementing the systems when there are substantial staff shortages and staff turnover. Such shortages meant that job duties and roles changed regularly, as managers shifted staff around to cover gaps. Additionally, others noted that the system helped with reminders for when patients were due for viral load testing, and some pointed out the potential for the system to help with patient follow-up across clinics if the system were to be implemented nationally or even regionally.

Several clinicians talked about how the mHealth system benefitted patients during the study, and discussed how the mHealth system had the potential to benefit all patients in the clinic were the system to be implemented broadly. Specifically, they noted that the system helped patients transition from every month prescription pick-ups to 3-month prescription pick-ups as it helped with visit attendance and thus with retention in care.

Others recognized that the system helped patients come to appointments on schedule and remarked that the system led to more complete viral load testing as it helped to reduce loss to follow-up. Finally, some clinicians recognized that the system had positive spillover effects even for those who were not enrolled in the study.

…because the system is there and there is a close follow up, it helps to remind us that the patient is required to conduct a test, it has motivated us and increased our attention in making follow up, not only for those who are in the system but also for all patients in general. (CTC In Charge, clinic B)

### Adoption

#### Patient perspective

To measure adoption, we explored the proportion of PLHIV study participants who had their mobile bank accounts linked to the mHealth system at the end of the study. At the end of the study, 88% of study participants reported that they had access to a mobile phone, 78% reported that they had access to a mobile money account, and among those in the cash groups, 81% had registered their mobile money account with the mHealth system.

#### Clinical staff perspective

We measured adoption from the clinical staff perspective by examining the proportion of time that the pharmacist was operating the mHealth system — the intended design. As the mHealth system was rolled out in the clinics, research assistants provided significant levels of support to the pharmacist in registering patients with the mHealth system; however, by the end of the study, the pharmacist was operating the system on average 72% of the time (as opposed to the research assistant being the primary operator).

### Fidelity

#### Patient perspective

Among PLHIV in the cash award groups (*n* = 346), 331 (96%) received at least one cash transfer during the 6-month study, and the average number of cash transfers per study participant was 4.7 (out of a possible 6 transfers). Out of a total of 1651 cash transfers delivered to study participants, 1283 (78%) were delivered through mobile money (the remainder were paid to participants in cash) and 2.5% of those sent through mobile money required manual re-sending due to network failures.

#### Clinical staff perspective

To assess fidelity of implementation from the clinical staff perspective, we looked at the proportion of visits for which the mHealth system was used. Over 3067 total clinical visits across all four health facilities during the 6 months following study enrollment, 172 (5.6%) were not captured in the mHealth system; 94.4% of all visits were registered into the mHealth system at the time of visit. The proportion of visits for which the mHealth system was used ranged from 94.6 to 89.1% by clinical site.

### Feasibility — clinical staff perspective

**Fig. 1:**
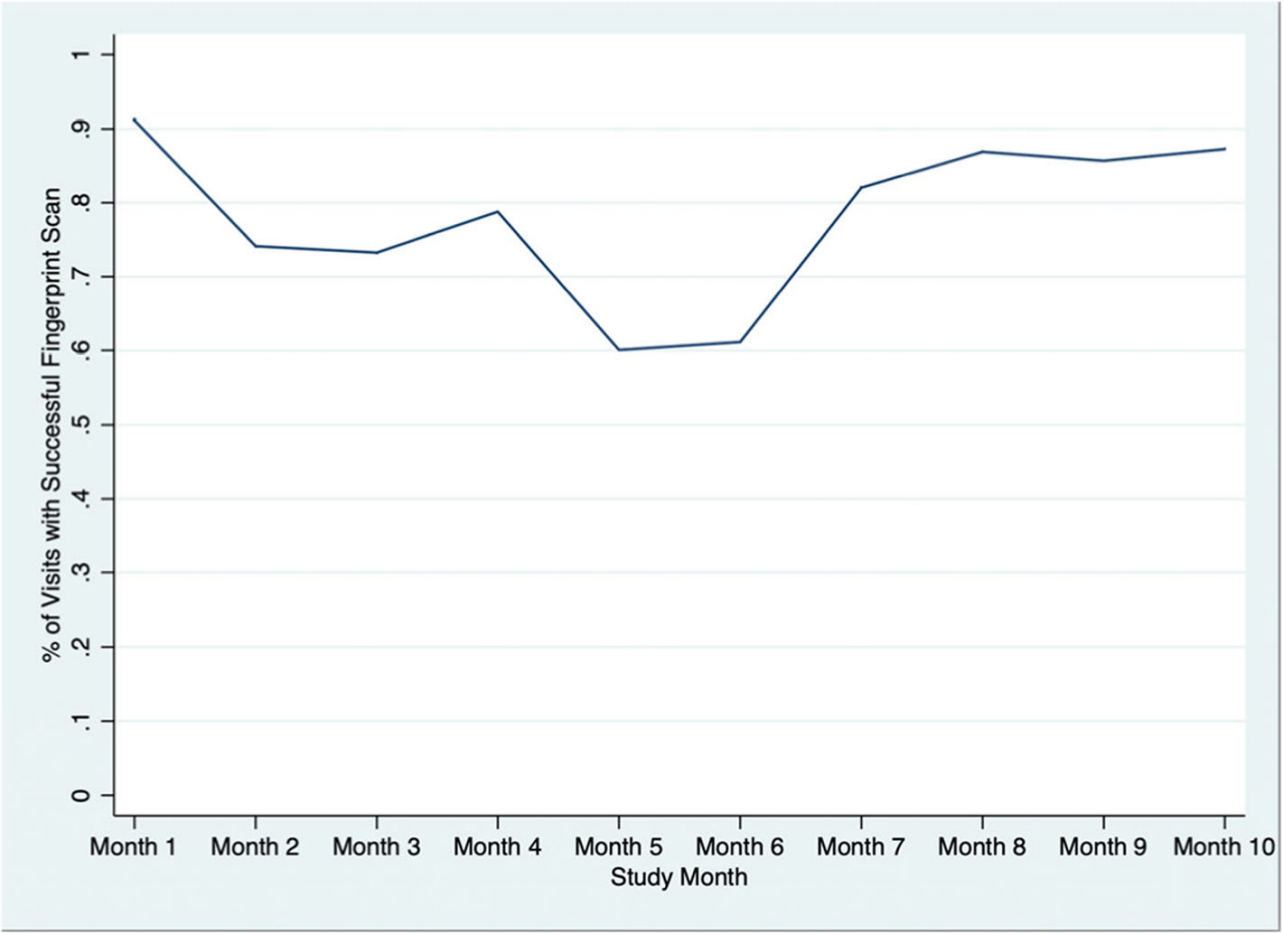
Fingerprint scan success rate, by study month

To assess feasibility, we focused on the technological aspects of the mHealth system implementation, exploring how frequently the fingerprint scanning system and mobile money distribution systems failed. The clinic pharmacy structured observations captured data on 2293 patient visits over the course of the study. Observations were focused on the use of the mHealth system during clinic visits and included the number of fingerprint scans required until the mHealth system successfully identified the patient, and whether or not the fingerprint scan was eventually successful in identifying the patient (regardless of the number of scans required). It should be noted that as more PLHIV enrolled in the study and more fingerprints were added to the database, finding correct fingerprint matches became more complex and required several iterations of the matching algorithm. Overall, fingerprint recognition succeeded for 74.1% of visits while 25.9% required manual entry of the patient's unique identification number due to poor image quality. The success rate for fingerprint recognition increased over time; by the final month of structured observations, the success rate was 87.3% (Fig. 1). Overall, the average number of fingerprint scans required for the mHealth system to successfully identify the patient was 2.04; this also varied considerably by study month, and by the end of the study, the average number of scans was 1.8.

### Sustainability — clinical staff perspective

In an effort to explore scale-up and sustainability, we asked the clinicians about what challenges they might anticipate encountering should the mHealth system offset some paper-based aspects of the current recordkeeping system. Many of the themes that emerged had already been mentioned, including the importance of training, considerations related to staff turnover, and the need for a salary top-up as many staff saw use of the system as an added task rather than as a way to simplify or facilitate their existing work.

The potential benefit for patients was another theme that arose when discussing sustainability of the mHealth system. Several clinicians noted recognized the potential benefits and mentioned that with adequate training, they would be prepared for the new system.

We are positively ready for it [mHealth system] as I have said that we have observed its high impact within this short time of using it…we have observed the positive impacts so as the clinic we are ready for the changes which will come. (CTC In Charge, clinic B)

## Discussion

To our awareness, this is the first cash transfer program designed to improve retention in care that has been implemented through an mHealth system [20]. In general, we found that the mHealth system was overall successfully implemented with high levels of acceptance and usability from both patients and providers; the system we designed was implemented with a high degree of fidelity and functioned as intended. While fingerprint recognition encountered some feasibility limitations in the first months of testing, payments via mobile money were highly successful. Clinic management staff voiced excitement for the system’s potential to bring the cash program to patients and simplify workflows; yet concerns about multiple systems, staffing shortages, and intermittent connectivity tempered enthusiasm, highlighting structural issues beyond the scope of the program.

Still, there were unintended benefits and consequences, many of which could impact scale-up efforts. Specifically, fingerprint identification plus automatic mobile payments have strong potential as a means to efficiently implement clinic-based cash incentives in low-income country settings. In fact, utilization of mobile money has increased significantly in low-resource settings (over 50% of Tanzanians have a mobile money account), and continues to grow quickly [34]. As a result, social protection programs in low-resource settings that utilize cash transfers are increasingly using mobile money instead of cash due to its multiple advantages, including the potential for increasing financial inclusion, decreased travel and time requirements for recipients, and increased accuracy and accountability [35, 36]. However, based on our results, the following should be considered: (a) upfront effort needs to be expended to ensure that patient concerns about privacy are addressed, for example by allowing participants to opt-out of SMS notifications, or by providing assurance that SMS messages do not reveal anything about HIV status; (b) while most participants had access to mobile phones and mobile money, providing access for all will be needed to successfully implement this system and consideration given to how to weigh this need against the ability to bring the intervention to scale; (c) implementation support in the clinic including universal training, incentive payments for staff to use the system, and ensuring there is a solid understanding of the benefits conferred to patients (e.g., decreasing loss to follow-up) will be crucial; (d) getting the fingerprint system right took more time and was more complicated than expected — issues relating to image quality, computing storage, and creating the threshold precision level for fingerprint recognition resulted in a steep learning curve; and (e) unexpected benefits of the system included lending a sense of legitimacy and belonging for patients, a strong understanding among clinical staff of how such a system could help patients and simplify clinic operations, and as a result of the mobile money, the potential for increased savings and increased control over spending, as well as increasing intrahousehold bargaining power for women. These findings are supported by other research and data showing that mobile money payments (as compared to cash) provide an early entry point into the formal financial sector and provide the opportunity for women to have more control over their money and how they spend it [37–39].

It should be noted that some characteristics inherent to many clinical settings in resource constrained settings may limit the impact of any mHealth system, and thus, impact the potential for scale and sustainability. Many of these were mentioned by clinical staff; these include infrastructure (reliable Wi-Fi networks, replacement tablets, secure storage), staffing shortages and turnover, and the need for universal training and sustained training support for using the system. These inherent limitations underscore the need for developing a system that is easy to use, is scalable, and does not rely on paper-based tracking systems and manual identification of eligible beneficiaries for a cash transfer program. In addition, working with a local technology firm who understands well the context and unique challenges that arise in these settings is essential.

This study has some limitations. Gaining an understanding of how this mHealth system functions outside of the study context is challenging, particularly with significant research staff involvement during implementation. We explored this by assessing the proportion of visits that were handled by the pharmacist versus the research staff at study end; these data showed a trend toward implementation as designed, with the pharmacist operating the system. Additionally, we will have the opportunity to explore this aspect of implementation in more detail during phase 2 of our study [28], an ongoing cluster RCT in 32 clinics. This will provide ample opportunity to assess heterogeneity in implementation and its impact on the primary study outcome [28]. Additionally, in-depth interviews conducted with a small subsample of study participants may not be representative of the full study population, and some data collection methods (e.g., structured observation in the clinics and the HITUES questions) were introduced toward the end of the study, and may not represent the full study experience. Social desirability bias may impact reported perceptions of the mHealth system, and the lack of data from PLHIV participants in the cash groups who received cash payments (rather than mobile money) may be a limitation. To mitigate some of these concerns, we triangulated data from multiple sources (surveys, interviews, clinic observation).

## Conclusions

Biometric identification and mobile payments may provide a scalable mechanism to efficiently implement cash incentives in low-income country settings, and mobile money payments may result in the unintended benefit of increasing savings and providing a means to control spending. An ongoing cluster RCT evaluating the cash incentive intervention described herein will further explore implementation outcomes, with a particular focus on scalability and sustainability [28].

## Supplementary Information

**Additional file 1 **: **Table 1**. HITUES domains and questions as adapted for the mHealth system.

## Data Availability

The datasets used and analyzed during the current study are available from the corresponding author on reasonable request.

## Abbreviations

PLHIV: People living with HIV/AIDS
ART: Antiretroviral therapy
RCT: Randomized controlled trial
IDI: In-depth interview
HITUES: Health Information Technology User Evaluation Scale
WHO: World Health Organization
