# Supplementary material for: Preparing a financial incentive program to improve retention in HIV care and viral suppression for scale: using an implementation science framework to evaluate an mHealth system in Tanzania
Source: Implement Sci Commun. 2021 Sep 23;2:109. doi: 10.1186/s43058-021-00214-w (PMC8461932; doi:10.1186/s43058-021-00214-w)
Supplement: Supplementary file 1 — Additional file 1 : Table 1. HITUES domains and questions as adapted for the mHealth system. [file 43058_2021_214_MOESM1_ESM.docx]

**Supplemental Material**

**Table 1:** HITUES domains and questions as adapted for the mHealth system

| **Domain** | **HITUES Questions – Cash Disbursement** | **HITUES Questions – Biometric ID** |
| --- | --- | --- |
| Impact | Compared to receiving cash in hand, I think automatic mobile money is a positive addition for persons living with HIV  Compared to cash in hand, I think automatic mobile money improves the quality of life of persons living with HIV  Compared to cash in hand, automatic mobile money is an important part of meeting my needs related to staying in HIV care and being adherent to my medication | I think Fingerprint ID is a positive addition for persons living with HIV  I think Fingerprint ID improves the quality of life of persons living with HIV  Fingerprint ID is an important part of meeting my needs related to retention and adherence to my medication |
| Usefulness | Using automatic mobile money (compared to getting cash in hand) makes it easier for me to find time to come to the clinic to pick up my medication  Using automatic mobile money (compared to getting cash in hand) makes it easier for me to find money to come to the clinic to pick up my medication  Having my money transferred to me automatically, rather than receiving cash in hand, enables me to stay adherent to my medication  Using automatic mobile money, rather than receiving cash in hand, makes it more likely that I will pick up my medication on time  Using automatic mobile money compared to receiving cash in hand is useful for getting the cash transfer quickly and easily  I am satisfied with automatic mobile money for getting my cash transfer  I can receive my money in a more timely manner because of automatic mobile money  Using automatic mobile money (compared to receiving cash in hand) increases my ability to make it to my clinic appointments | Using Fingerprint ID makes it easier for me to pick up my medication  Using Fingerprint ID enables me to quickly receive my cash transfer  Using Fingerprint ID is useful for the clinic to correctly identify me  I am satisfied with Fingerprint ID for helping me to get my medication  I can pick up my medication in a more timely manner because of Fingerprint ID  Using Fingerprint ID increases my ability to complete my visit efficiently? |
| Ease of Use | I am comfortable with my ability to use automatic mobile money  Figuring out how to use automatic mobile money is easy for me  It is easy for me to become skilled at using automatic mobile money  I find automatic mobile money easy to use  I can always remember how to use automatic mobile money | I am comfortable with my ability to use Fingerprint ID  Learning to operate Fingerprint ID is easy for me  It is easy for me to become skilled at using Fingerprint ID  I find Fingerprint ID easy to use |
| User Control | Automatic mobile money gives error messages that clearly tell me how to fix problems  I am sometimes concerned that the automatic mobile money payment won’t work and I will end up with no money  The information provided on how to use automatic mobile money is clear | Someone may hack into the Fingerprint ID system and steal my personal information  I trust the Fingerprint ID system  I am concerned that my personal data in the system is shared with third parties without my agreement  I feel confident that the Fingerprint ID will keep information about my HIV status confidential  I feel confident that the Fingerprint ID will keep information about my mobile money payment confidential  I feel confident that the Fingerprint ID will keep data about me and my clinic visit secure  The information provided about Fingerprint ID and why it is being used is clear |
